# Developing and Testing a Brief Mindfulness Just-in-Time Adaptive Intervention to Reduce Stress Among Caregivers of People With Dementia: Quasi-Experimental Study

**DOI:** 10.2196/87316

**Published:** 2026-06-05

**Authors:** Patrick Pui Kin Kor, Alex Pak Lik Tsang, Daphne Sze Ki Cheung, Steven H Zarit, Haoran Xie, Min Qian, Kay Chen Tan, Yuanqing Zheng, Amanda Man Ying Chu, Kee Lee Chou

**Affiliations:** 1 School of Nursing, Faculty of Health and Social Sciences, Hong Kong Polytechnic University, Hung Hom, Kowloon, China (Hong Kong); 2 School of Nursing and Midwifery, Faculty of Health, Deakin University, Melbourne, Victoria, Australia; 3 Centre for Quality and Patient Safety Research/Alfred Health Partnership, Institute for Health Transformation, Deakin University, Melbourne, Victoria, Australia; 4 Department of Human Development and Family Studies, Pennsylvania State University, University Park, PA, United States; 5 Division of Artificial Intelligence, School of Data Science, Lingnan University, Tuen Mun, New Territories, China (Hong Kong); 6 Mailman School of Public Health, Columbia University, New York, NY, United States; 7 Department of Data Science and Artificial Intelligence, Faculty of Computer and Mathematical Sciences, Hong Kong Polytechnic University, Hung Hom, Kowloon, China (Hong Kong); 8 Department of Social Sciences and Policy Studies, Faculty of Liberal Arts and Social Sciences, Education University of Hong Kong, 10 Lo Ping Road, Tai Po, New Territories, China (Hong Kong), 852 29487473

**Keywords:** dementia caregiving, mindfulness, mobile health, just-in-time adaptive intervention, receptivity, stress, Android app

## Abstract

**Background:**

Dementia caregiving entails chronic, fluctuating stress with downstream risks to caregivers’ mental health and quality of care. Mindfulness-based interventions can reduce caregiver stress; however, moment-to-moment fluctuations in stress may limit receptivity to practice at any given time. We developed a brief mindfulness just-in-time adaptive intervention (JITAI) that aims to deliver support at the right moment by using machine learning algorithms to optimize notification timing based on receptivity to engage in brief mindfulness practices.

**Objective:**

This study aims to evaluate the feasibility, acceptability, and effectiveness of a brief mindfulness JITAI for caregivers of people with dementia on stress, depressive symptoms, caregiver burden, sleep, quality of life, and trait mindfulness.

**Methods:**

A single-arm, pretest or posttest design was adopted. A total of 120 community-dwelling caregivers were recruited to participate in the 18-day intervention, which included 4 days of psychoeducation delivered via videos and phone coaching, alongside an in-app brief, low-dose mindfulness-based stress reduction component. From days 5 to 11, prompts were delivered either by a static machine learning model trained on prior pilot data or at random times, with equal probability. From days 12 to 18, three delivery models were used with equal probability, namely static, random, and adaptive models, which updated per participant using accumulating receptivity data. Feasibility and acceptability were assessed post intervention. Standardized measures of stress, depressive symptoms, caregiver burden, positive aspects of caregiving, sleep, quality of life, and trait mindfulness were collected via phone interviews at baseline and post intervention.

**Results:**

Retention was 100%. Most participants (111/120, 92.5%) found the app easy to use, 81.7% (98/120) perceived it as helpful for stress management, and 80% (96/120) would recommend it to other caregivers. Pre-post analyses indicated significant reductions in perceived stress (*P*<.001), depressive symptoms (*P*<.001), and caregiver burden (*P*=.003), as well as a significant increase in positive aspects of caregiving (*P*<.001) and subjective sleep quality (*P*=.02). Health-related quality of life and trait mindfulness did not change significantly.

**Conclusions:**

A brief, smartphone-delivered mindfulness JITAI for caregivers of people with dementia was feasible and acceptable, with high retention and positive user evaluations. Pre-post findings suggest reductions in perceived stress, depressive symptoms, and caregiving burden, alongside increased positive aspects of caregiving and improved sleep, supporting the potential of adaptive, technology-enabled interventions to provide timely support to caregivers.

## Introduction

Dementia is a neurodegenerative condition characterized by progressive declines in functional abilities and substantial care needs, with caregiving often provided by family members. With an aging global population, the prevalence of dementia is projected to rise from 55 million in 2019 to 139 million by 2050 [1]. Consequently, the number of family caregivers assuming these demanding roles will increase. The stress associated with dementia caregiving is substantial and can lead to adverse health outcomes for both caregivers and care recipients. For caregivers, prolonged exposure to stress is linked to insomnia, compromised immune function, and psychological distress, including depression and anxiety [2-4]. For care recipients, high levels of caregiver stress can increase the likelihood of premature institutionalization and, in some cases, elder abuse [5,6]. Therefore, developing effective strategies to mitigate caregiver stress is a pressing public health priority.

Since stressors are a normative and unavoidable part of the long-term dementia caregiving trajectory, emotion-focused coping approaches that emphasize the positive reappraisal of caregiving-related emotions, in contrast to problem-focused approaches that target modification of the stressor itself (eg, caregiving skills training), are particularly important for stress management [7]. Mindfulness is one such approach and has been shown to reduce stress among caregivers of people with dementia [8,9]. The mindful coping model emphasizes decentering, defined as the capacity to observe one’s thoughts and feelings from a detached perspective [10]. This shift in attentional focus enables caregivers to reframe stressors, engage in positive reappraisal, and ultimately reduce stress. A systematic review has confirmed that mindfulness-based interventions (MBIs) have a moderate effect size in reducing caregiver stress in this population [8].

Despite their effectiveness, MBIs for caregivers of people with dementia are hindered by practical challenges. A primary barrier is the significant time commitment required by traditional program structures. For example, a trial of mindfulness-based cognitive therapy found that about 20% of 113 participants discontinued regular mindfulness practice during follow-up, with lack of time being the most common reason for discontinuing their practice [9]. Caregivers often juggle numerous caregiving tasks and other life commitments, which can disrupt the self-directed practice central to these interventions. The fluctuating nature of caregiving demands and stress can also create episodes of cognitive interference, making it difficult to focus attention during periods of high strain [11]. If an intervention is perceived as too intensive or inflexible, caregivers may be less motivated to engage in self-practice. Therefore, tailoring the delivery of mindfulness practices is crucial to support sustained engagement. To address this challenge, interventions should be delivered at moments of optimal receptivity, defined as states in which a caregiver is able, available, and willing to receive, process, and use support [12].

A just-in-time adaptive intervention (JITAI) can optimize receptivity by delivering support at opportune moments while adapting to a user’s changing internal and external contexts [12]. This approach has been applied to a wide range of public health issues, including physical inactivity [13], smoking cessation [14], and obesity [15]. More recently, JITAIs have been integrated with MBIs for individuals with chronic pain, demonstrating reductions in cravings, pain, and stress, along with increases in positive affect [16]. Receptivity in JITAI delivery can be optimized using several models: (1) static, (2) random, and (3) adaptive [17]. A static model delivers prompts at predetermined times, often derived from machine learning analyses of prior user data; however, receptivity patterns from one group may not generalize to others [18]. A random model delivers prompts at random times and often underperforms machine learning–based models [17]. An adaptive model uses machine learning to learn each participant’s receptivity patterns as the study progresses. Prior research suggests that although an adaptive model may underperform initially, its performance improves as user-specific data accumulate over time [17].

Taken together, a brief mindfulness-based JITAI may offer a promising approach to the unique challenges of delivering support to this population. By tailoring delivery to moments of high receptivity, a JITAI can facilitate the self-directed practice essential for maximizing the stress-reduction benefits of MBIs [19]. However, there is limited research applying mindfulness-based JITAIs to caregivers of people with dementia. Consequently, their effectiveness for stress reduction, as well as their feasibility and acceptability within this population, remains unclear.

This study aims to address this gap by introducing and evaluating a novel, brief mindfulness JITAI for caregivers of people with dementia using Android phones. The study objectives are (1) to evaluate the feasibility and acceptability of the intervention (eg, retention, adherence or engagement with prompted practice, and user feedback) and (2) to estimate preliminary pre-post changes in caregiver psychosocial outcomes (eg, perceived stress and related outcomes). To operationalize these objectives and manage the initial learning phase of the adaptive model, the program was implemented in the following steps: step 1 (1‐4 d) provided brief psychoeducation and coaching with access to app-based mindfulness content; step 2 (5‐11 d) delivered prompts using either a static model informed by pilot usage data (n=75) or a random model (equal probability) to accumulate initial data; and step 3 (12‐18 d) introduced an adaptive model (alongside static and random, equal probability) that personalized prompt timing based on continuously collected, participant-specific data.

## Methods

### Study Design

This is a single-arm, quasi-experimental study. The study is reported in accordance with the CONSORT (Consolidated Standards of Reporting Trials) guidelines (Checklist 1). It was conducted in compliance with the Declaration of Helsinki (and its subsequent amendments) and Good Clinical Practice guidelines.

### Ethical Considerations

Ethical approval was obtained from the corresponding author’s institution (reference: A2021-2022-0276-01). All participants provided written informed consent prior to participation. Participants were informed of the study’s purpose, procedures, the voluntary nature of participation, and their right to withdraw at any time without penalty. Privacy and confidentiality were protected by deidentifying participant data and restricting access to research data to authorized members of the research team only. Participants who completed all assessments and attended at least 80% of the intervention sessions received HK $500 (US $64.10) in supermarket coupons as compensation for their time and participation. The trial was registered at the Chinese Clinical Trial Registry (ChiCTR2300071361).

### Participants

Participants were family caregivers of people with dementia, recruited through a network of nongovernmental organizations providing community services for older people, caregiver support groups, and the Alzheimer’s Association in Hong Kong.

Eligibility criteria included being aged 18 years or older, serving as a family caregiver for a community-dwelling individual with a confirmed medical diagnosis of any type of dementia, providing at least 20 hours per week of assistance with activities of daily living and instrumental activities of daily living for a minimum of 1 year prior to recruitment, having an Android smartphone, and reporting moderate to high stress as measured by the 14-item Perceived Stress Scale (PSS-14) [20]. A cutoff score of 18 was used to identify caregivers with elevated stress (PSS-14>18), consistent with thresholds applied in previous studies [21].

Exclusion criteria included participation in any structured mind-body intervention, cognitive behavioral therapy, or structured psychosocial intervention within the previous 6 months, as well as acute psychiatric or medical comorbidities that were potentially life-threatening (eg, suicidal ideation) or could limit participation or adherence (eg, acute psychosis).

### Procedures

#### Recruitment and Assessment

Interested individuals were approached by a research assistant, who administered a brief screening questionnaire via phone interviews to confirm eligibility. Those who met the inclusion criteria received a standardized study briefing and then provided electronic informed consent via the Qualtrics platform (Qualtrics International Inc). The research team carefully explained the required commitments to ensure that participants were aware of expectations and to minimize attrition during the study period.

Data were collected through structured telephone interviews conducted by a trained research assistant at baseline (T1; the first day of the study period) and post intervention (T2; the last day of the study period).

#### The Brief Mindfulness JITAI

**Figure 1.**
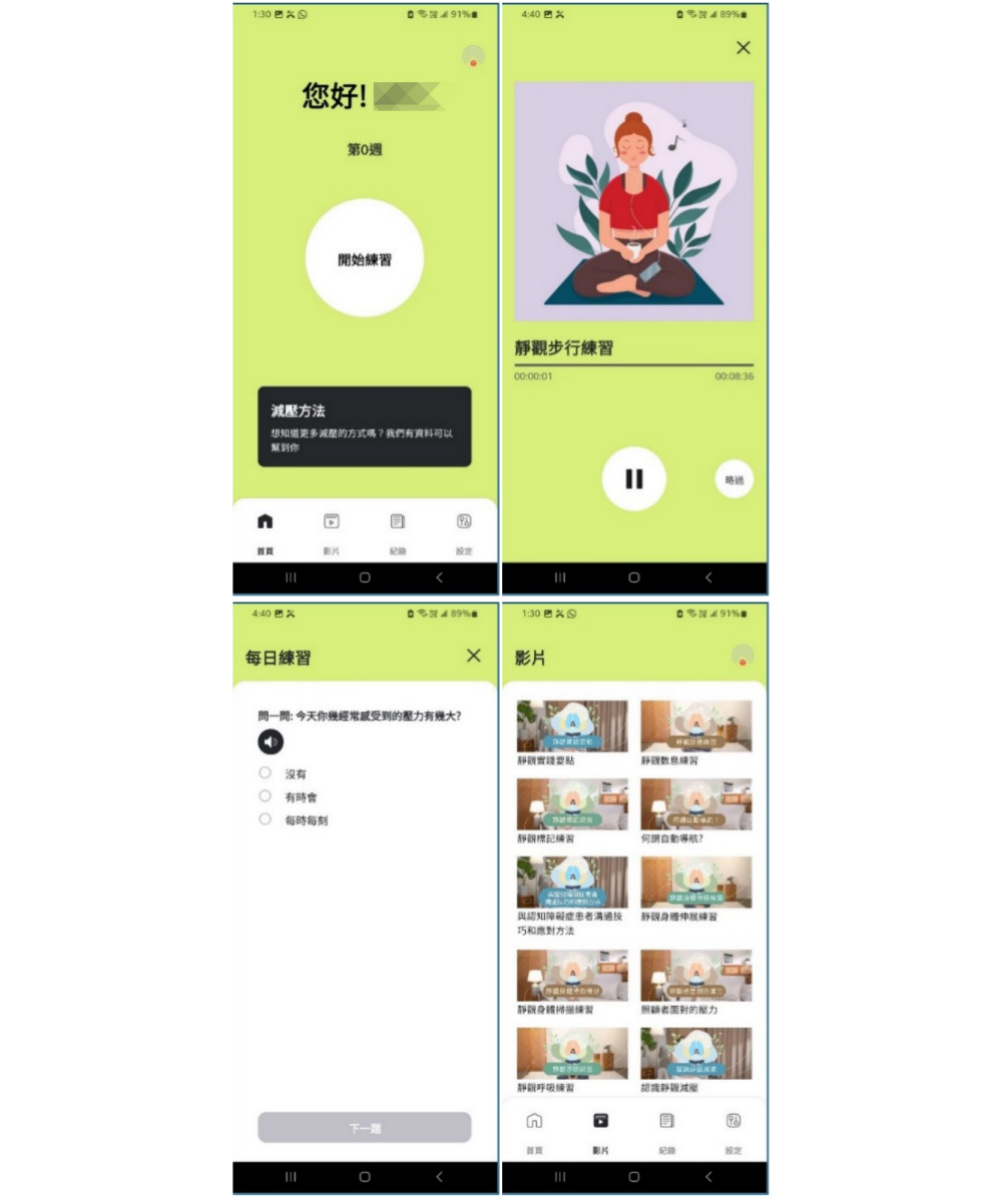
Screenshots of the mobile app. The top-left panel shows the home screen, which greets the user (“您好”) and prompts them to start practice (“開始練習”) for week 0 (“第0週”). The top-right panel displays guided practice for a mindful walking exercise (“靜觀步行練習”), with a progress bar and playback controls. Participants can skip the exercise by pressing “略過.” The bottom-left panel features a check-in function (“每日練習”) that asks users to rate their stress level for the day (“今天你經常感受到的壓力有多大？”). The bottom-right panel showcases the video library (“影片”), which contains modules such as “Communication Skills with Dementia Patients” (“與認知障礙症患者溝通技巧和應對方法”) and “Stress Faced by Caregivers” (“照顧者面對的壓力”).

The brief mindfulness JITAI lasted 18 days. During days 1 to 4, participants received daily 30-minute psychosocial education sessions comprising short videos and phone-based coaching, alongside access to a brief, app-embedded mindfulness program (Figure 1). The study app was distributed to participants as an Android Package Kit for installation and was not publicly available via commercial app stores at the time of the study. Intervention content was developed by a certified mindfulness instructor and drew on “low-dose” mindfulness-based stress reduction (MBSR) elements [22]. MBSR was selected given its demonstrated feasibility, acceptability, and benefits for stress reduction among family caregivers of people with dementia [8,23,24]. Beginning on day 5, participants received push notifications prompting mindfulness practice.

**Figure 2.**
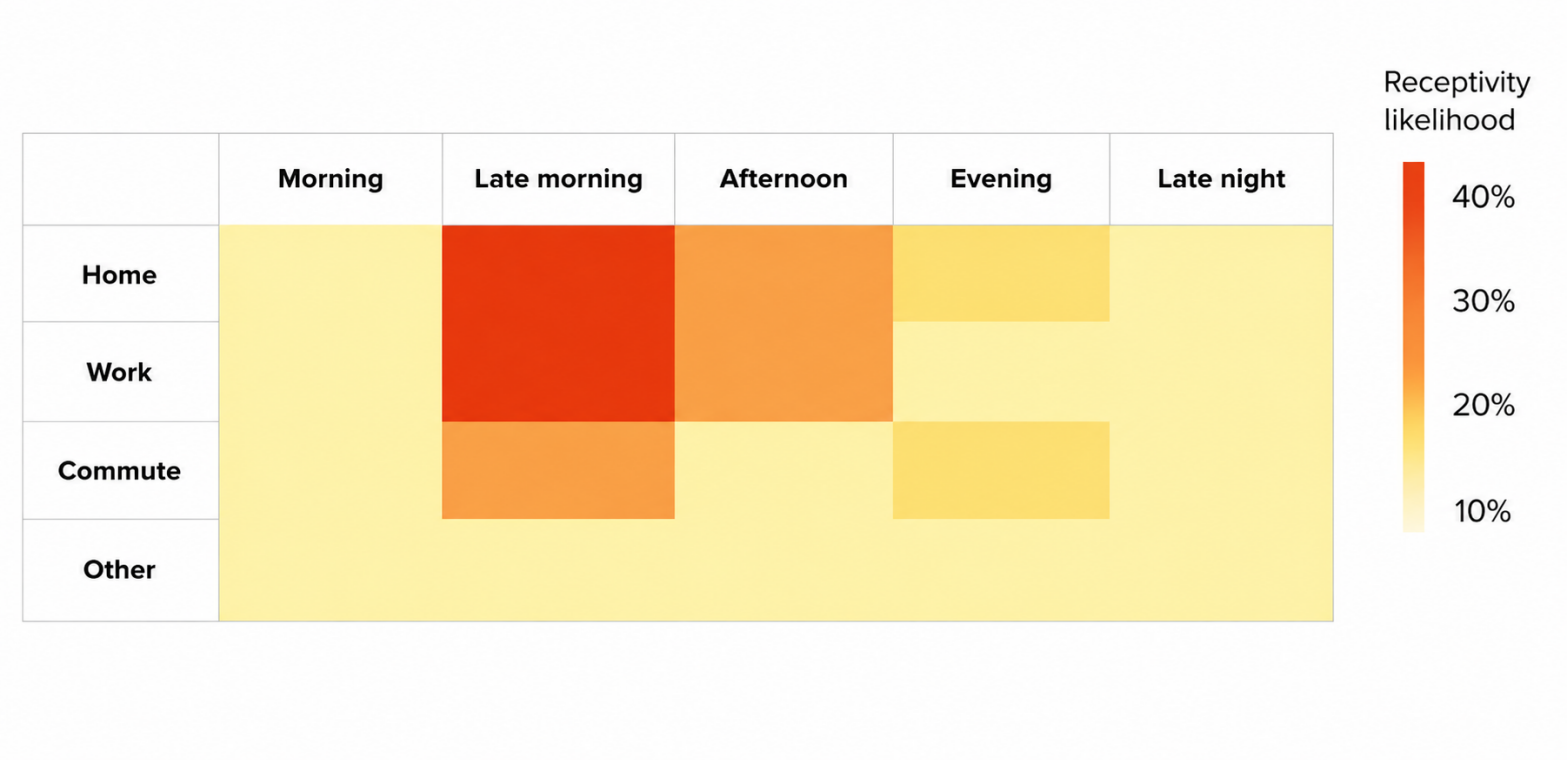
Receptivity likelihood heatmap for training the static model.

From days 5 to 11, the timing of intervention prompts was determined either by (1) a static machine learning model trained in a pilot study with 75 participants using 4 weeks of passive smartphone sensing data (day of week, time of day, type of day [weekday vs weekend], battery level, and location) and observed receptivity, operationalized as the just-in-time response rate (ie, whether a delivered notification prompted engagement with the mindfulness practice within 10 min of delivery), consistent with definitions used in prior JITAI studies [17] (Figure 2), or (2) random scheduling with equal probability. The static model remained fixed across all participants and days.

From days 12 to 18 (post–warm-up period), an adaptive model was added in addition to the static and random models. The adaptive model updated its predictions as the study progressed using each participant’s accumulating observed receptivity data (just-in-time response rate) and the same contextual feature set used in the static model. We introduced the adaptive model after 1 week to ensure that sufficient participant-specific data had been accumulated to inform model predictions.

Decision logic for notification timing was as follows: the static and adaptive models each used a classifier to infer whether the current moment was “receptive” for delivering a prompt; the random scheduler did not screen for receptivity and always approved delivery. If a model judged the current moment as not receptive, the app queried the same model again every 5 minutes. If no receptive moment was inferred within 30 minutes, a notification was sent, and this delivery was recorded under the random model.

### Measures

#### Feasibility Outcomes

Intervention feasibility was assessed using recruitment, attendance, completion, and retention metrics. The recruitment rate was defined as the proportion of eligible individuals who enrolled. The attendance rate reflected the proportion of the 4 psychoeducation sessions attended. The completion rate referred to the proportion of participants who finished all study assessments. The retention rate represented the proportion of enrolled participants who remained throughout the study period.

#### Acceptability Outcomes

Intervention acceptability was assessed at T2 using a questionnaire with three sections: (1) acceptability of the mobile app, (2) acceptability of notifications, and (3) acceptability of mindfulness activities.

In the app section, participants indicated whether the app was easy to install and use (yes or no), whether they ignored the app’s notifications (yes or no), whether the app helped them manage stress (able or helpful or not able or not helpful), and whether they would recommend the app to other caregivers (would or would not).

In the notifications section, participants rated the number of messages (too many, appropriate, or too few) and the helpfulness or appropriateness of their delivery timing (helpful, appropriate, or not helpful). They also reported whether they liked the wording of the reminders (yes or no).

In the mindfulness activities section, participants indicated whether the activities helped them manage stress (able or helpful and not able or not helpful), whether they liked the graphics and characters used (like or do not like), and whether learning mindfulness through these activities was helpful (helpful or not helpful).

#### Effectiveness Outcomes

The primary outcome was perceived stress, measured using the PSS-14 [20]. Items are rated on a 5-point Likert scale (0=“never” to 4=“very often”), with higher scores indicating greater perceived stress. Cronbach α for the Chinese PSS-14 was 0.72 at T1 and 0.82 at T2.

Depressive symptoms were assessed using the 9-item Patient Health Questionnaire, which measures the frequency of depressive symptoms over the past week [25]. Items are rated on a 4-point scale (0=“not at all” to 3=“nearly every day”), with higher scores reflecting greater depression severity. Cronbach α for the Chinese Patient Health Questionnaire-9 was 0.91 at T1 and 0.89 at T2.

Caregiving burden was measured using the 12-item Zarit Burden Interview [26]. Items assess the perceived impacts of caregiving on emotional strain, physical fatigue, role restriction, and social well-being. Each item is rated on a 5-point scale (0=“never” to 4=“nearly always”), with higher scores denoting greater burden. Cronbach α for the Chinese 12-item Zarit Burden Interview was 0.86 at T1 and 0.87 at T2.

Positive caregiving appraisal was measured using the Positive Aspects of Caregiving Scale (PAC) [27]. The PAC assesses perceived gains from caregiving, including self-affirmation (a confident, capable self-image derived from the role) and outlook on life (interpersonal relationships and positive life orientation). Items are rated on a 5-point Likert scale (1=“strongly disagree” to 5=“strongly agree”), with higher scores indicating a more positive appraisal. Cronbach α for the Chinese PAC was 0.91 at both T1 and T2.

Subjective sleep quality and disturbances were assessed using the Pittsburgh Sleep Quality Index [28]. The Pittsburgh Sleep Quality Index aggregates component scores—subjective sleep quality, latency, duration, efficiency, disturbances, and daytime dysfunction—into a global score; higher scores indicate poorer sleep quality. Cronbach α was 0.69 at T1 and 0.67 at T2.

Health-related quality of life was measured using the EQ-5D-5L [29]. The EQ-5D-5L indexes problems across 5 domains (mobility, self-care, usual activities, pain or discomfort, and anxiety or depression), each rated on 5 levels (no, slight, moderate, severe, and extreme problems). A 0 to 100 visual analog scale records self-rated health. Higher scores reflect better health-related quality of life. Cronbach α for the Chinese EQ-5D-5L was 0.71 at T1 and 0.69 at T2.

Trait mindfulness was assessed using the 15-item Mindful Attention Awareness Scale, which measures the frequency of attention lapses and mindlessness in daily life [30]. Items are rated on a 6-point scale (1=“almost always” to 6=“almost never”), with higher scores indicating greater dispositional mindfulness. Cronbach α for the Chinese Mindful Attention Awareness Scale was 0.91 at T1 and 0.90 at T2.

#### Sociodemographic and Caregiving Characteristics

Caregivers’ sociodemographic characteristics included age, gender (male and female), educational level (primary or below, secondary, and tertiary), economic status (economically inactive and economically active), household size, household income, the use of government or organizational financial assistance (yes or no), and the presence of chronic illness (yes or no) and disability (yes or no). Care recipients’ sociodemographic characteristics included age, gender, chronic illness, and disability.

Caregiving characteristics included living arrangement (co-residing with the care recipient: yes or no), sole-caregiver status (yes or no), duration of caregiving (years), and weekly caregiving hours. The quality of the relationship between the caregiver and the care recipient was rated on a 5-point scale from “very good” to “very poor.”

### Statistical Analysis

Data were analyzed using IBM SPSS version 29. We first examined participants’ sociodemographic characteristics, followed by a descriptive assessment of feasibility and acceptability outcomes. Paired-samples *t* tests were then used to compare effectiveness outcomes from T1 to T2. Unless otherwise specified, all tests were 2-tailed with an α level of 5%.

We calculated the sample size using G*Power 3.1 (Heinrich Heine University Düsseldorf). The primary effectiveness outcome was perceived stress, with an effect size of *d*=0.33 drawn from a prior meta-analysis of MBIs for caregivers of people with dementia [31]. A 2-tailed pre-post analysis with an α level of .05 and 80% power indicated a required sample size of 75 to detect this effect. Allowing for 20% attrition, the target was increased by 15 participants, yielding a total of 90. Recruitment continued as time and resources permitted to further increase statistical power.

## Results

### Sociodemographic and Caregiving Characteristics

**Figure 3.**
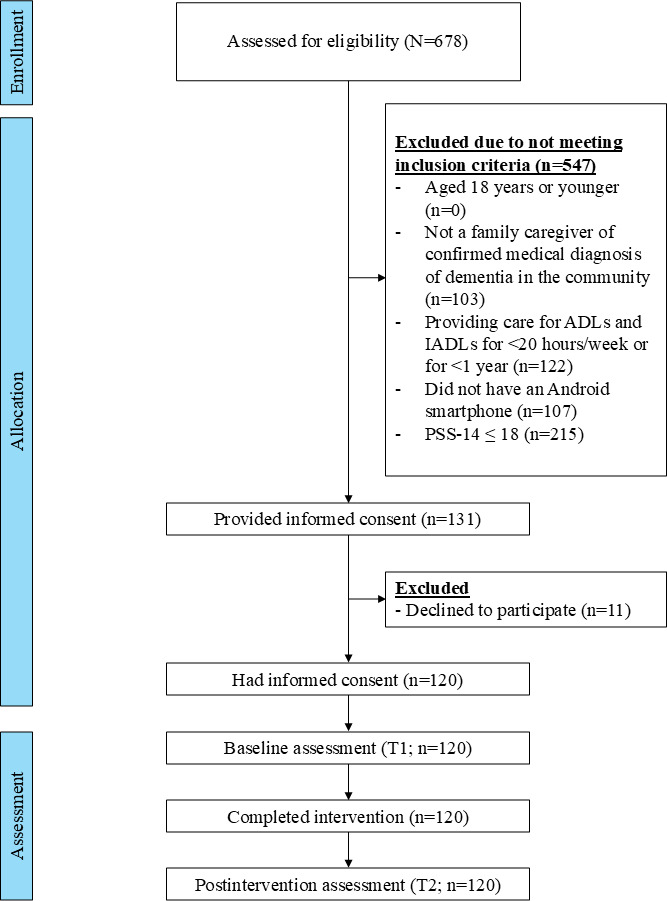
CONSORT (Consolidated Standards of Reporting Trials) flow diagram. ADL: activities of daily living; IADL: instrumental activities of daily living; PSS-14: 14-item Perceived Stress Scale.

A total of 120 caregivers participated in the study. A CONSORT flow diagram illustrating the participants’ progression is shown in Figure 3. Table 1 presents the participants’ sociodemographic and caregiving characteristics.

**Table 1.** Sociodemographic and caregiving characteristics (N=120).

Characteristics	Values
Caregivers’ characteristic	
Age (y), mean (SD)	45.4 (11.8)
Gender, n (%)	
Male	40 (33.3)
Female	80 (66.7)
Educational level^a^, n (%)	
Primary or below	13 (10.8)
Secondary	41 (34.2)
Tertiary	66 (55)
Employment status, n (%)	
Economically inactive	26 (21.7)
Economically active	94 (78.3)
Household size, mean (SD)	2.9 (0.9)
Household income (HK $; HK $1=US $0.128), mean (SD)	31,541.70 (20,079.10)
Use of government or organizational financial assistance, n (%)	
Yes	11 (9.2)
No	109 (90.8)
Presence of chronic illness, n (%)	
Yes	12 (10)
No	108 (90)
Presence of disability, n (%)	
Yes	0 (0)
No	120 (100)
Care recipients’ characteristics	
Age (y), mean (SD)	76.3 (7.4)
Gender, n (%)	
Male	54 (45)
Female	66 (55)
Presence of chronic illness, n (%)	
Yes	97 (80.8)
No	23 (19.2)
Presence of disability, n (%)	
Yes	20 (16.7)
No	100 (83.3)
Relationship with the caregiver, n (%)	
Mother	55 (45.8)
Father	44 (36.7)
Spouse	9 (7.5)
Paternal grandmother	7 (5.8)
Maternal grandfather	2 (1.7)
Paternal grandfather	1 (0.8)
Mother-in-law	1 (0.8)
Daughter-in-law	1 (0.8)
Caregiving characteristics	
Co-residing with the care recipient, n (%)	
Yes	84 (70)
No	36 (30)
Sole-caregiver status, n (%)	
Yes	43 (35.8)
No	77 (64.2)
Duration of caregiving (y), mean (SD)	5.5 (3.5)
Weekly caregiving hours, mean (SD)	45.9 (20.1)
Relationship with the care recipient, n (%)	
Very bad	0 (0)
Bad	2 (1.7)
Average	39 (32.5)
Good	58 (48.3)
Very good	21 (17.5)

a Tertiary education was defined as the completion of any postsecondary program, including subdegree (associate degree or higher diploma), bachelor’s degree, or postgraduate qualification.

Caregivers ranged in age from 18 to over 80 years, with a mean age of 45.4 (SD 11.8) years. Most were female (80/120, 66.7%), had completed tertiary education (66/120, 55%), and were economically active (94/120, 78.3%). The mean household size was 2.9 (SD 0.9) persons. The average monthly household income was HK $31,541.70 (SD HK $20,079.10), approximately US $4052.48. A minority of households (11/120, 9.2%) received financial assistance from the government or other organizations. Most caregivers reported having no chronic illness (108/120, 90%) and no disability (120/120, 100%).

Care recipients were older, with a mean age of 76.3 (SD 7.4) years, and the majority were female (66/120, 55%). Most had chronic health conditions (97/120, 80.8%), while a smaller proportion reported disabilities (20/120, 16.7%). The caregiving relationship was predominantly intergenerational. Most caregivers were caring for a parent, most commonly their mother (55/120, 45.8%) or father (44/120, 36.7%). Smaller proportions cared for a spouse (9/120, 7.5%) or other relatives, including grandparents and in-laws (12/120, 10% combined).

Regarding the caregiving characteristics, 70% (84/120) of caregivers lived with their care recipients. Approximately one-third (43/120, 35.8%) were identified as the sole caregiver. The duration of caregiving averaged 5.5 (SD 3.5) years. On average, caregivers devoted 45.9 (SD 20.1) hours per week to caregiving. The caregiver-care recipient relationship was generally positive: 48.3% (58/120) rated it “good” and 17.5% (21/120) “very good.”

### Feasibility Outcomes

Of the 131 eligible caregivers, 120 (91.6%) enrolled. Attendance, assessment completion, and retention were 120/120 (100%): all participants attended the 4-day psychoeducation sessions and completed both the T1 and T2 assessments, and no dropouts occurred during the study period.

### Acceptability Outcomes

Table 2 presents the acceptability outcomes of the brief mindfulness JITAI. Most participants (111/120, 92.5%) found the app easy to install and use. Although a notable proportion of participants reported ignoring notifications at times (68/120, 56.7%), the majority felt the app helped them manage stress (98/120, 81.7%) and would recommend it to other caregivers (96/120, 80%). Regarding notifications, most participants considered the number of messages appropriate (87/120, 72.5%), and a substantial proportion found the timing of the messages appropriate (52/120, 43.3%). The majority of the participants liked the wording of the reminders (107/120, 89.2%).

**Table 2.** Acceptability outcomes of the brief mindfulness JITAI^a^ (N=120).

Outcomes	Values, n (%)
Acceptability of the mobile app	
The app was easy to install and use	
Yes	111 (92.5)
No	9 (7.5)
Ignored the app’s notifications	
Yes	68 (56.7)
No	52 (43.3)
The app helped me manage stress	
Able or helpful	98 (81.7)
Not able or not helpful	22 (18.3)
Would recommend the app to other caregivers	
Would	96 (80)
Would not	24 (20)
Acceptability of notifications	
Adequacy of the number of messages	
Too many	27 (22.5)
Appropriate	87 (72.5)
Too few	6 (5)
Helpfulness or appropriateness of notification timing	
Helpful	47 (39.2)
Appropriate	52 (43.3)
Not helpful	21 (17.5)
Liked the wording of the reminders	
Yes	107 (89.2)
No	13 (10.8)
Acceptability of mindfulness activities	
Mindfulness helped me manage stress	
Able or helpful	102 (85)
Not able or not helpful	18 (15)
Liked the graphics and characters used	
Like	99 (82.5)
Do not like	21 (17.5)
Learning mindfulness through these activities was helpful	
Helpful	102 (85)
Not helpful	18 (15)

a JITAI: just-in-time adaptive intervention.

Participants also indicated that the mindfulness activities generally helped them manage stress (102/120, 85%), that they liked the app’s graphics and characters (99/120, 82.5%), and that the app was helpful for learning mindfulness (102/120, 85%).

### Effectiveness Outcomes

**Figure 4.**
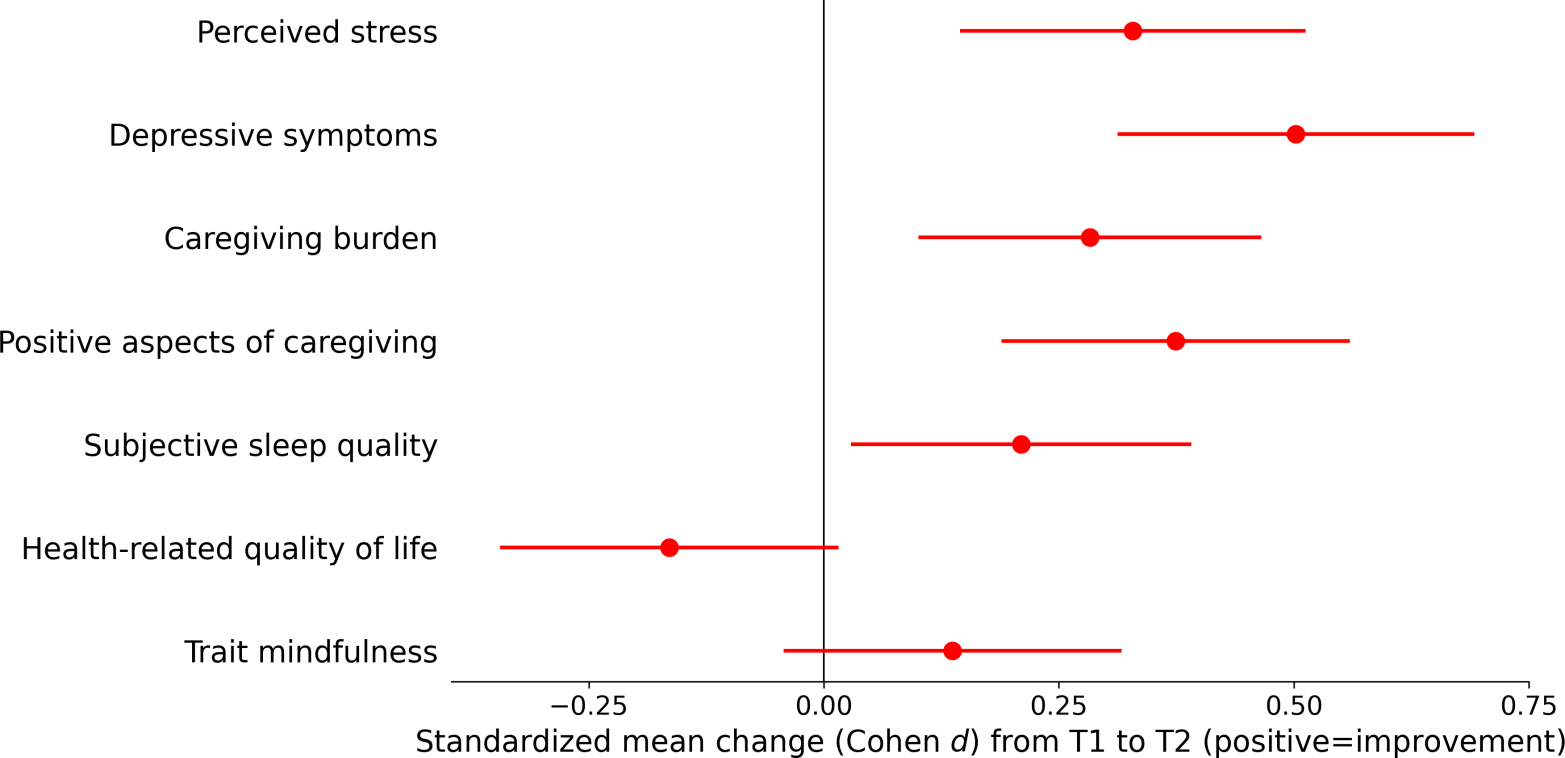
Forest plot of standardized outcome changes from T1 to T2. Positive values indicate improvement. For outcomes in which lower scores reflect better status (perceived stress, depressive symptoms, caregiving burden, and subjective sleep quality), effect sizes were sign-reversed so that positive values consistently represent improvement across measures. Error bars indicate 95% CIs.

Table 3 presents the effectiveness outcomes of the brief mindfulness JITAI. Figure 4 provides a visual summary of the standardized pre-post changes (effect sizes) across outcomes. The analysis of preintervention and postintervention effectiveness outcomes revealed statistically significant improvements across several psychosocial domains. Participants showed a significant reduction in perceived stress (*P*<.001), depressive symptoms (*P*<.001), and caregiving burden (*P*=.003). Conversely, there was a significant increase in the positive aspects of caregiving (*P*<.001). A significant improvement in subjective sleep quality (ie, scores decreased) was also observed (*P*=.02). However, no statistically significant changes were found for health-related quality of life (*P*=.08) or for trait mindfulness (*P*=.14).

**Table 3.** Effectiveness outcomes of the brief mindfulness JITAI^a^.

Outcomes	Baseline (T1), mean (SD)	Postintervention (T2), mean (SD)	*t* (*df*)	*P* value	*d*
Perceived stress	30.1 (3.8)	28.6 (4.8)	–3.6 (119)	<.001	–0.33
Depressive symptoms	8.9 (5.1)	6.7 (4.4)	–5.5 (119)	<.001	–0.50
Caregiving burden	22.8 (5.5)	21.1 (5.9)	–3.1 (119)	.003	–0.28
Positive aspects of caregiving	35.7 (6.5)	38.1 (6.3)	4.1 (119)	<.001	0.37
Subjective sleep quality	6.6 (2.8)	6.2 (2.6)	–2.3 (119)	.02	–0.21
Health-related quality of life	0.9 (0.1)	0.8 (0.1)	–1.8 (119)	.08	–0.16
Trait mindfulness	59.2 (9.2)	60.3 (9.1)	1.5 (119)	.14	0.14

a JITAI: just-in-time adaptive intervention.

## Discussion

### Principal Results

This study evaluated a brief mindfulness JITAI for family caregivers of people with dementia and found it to be a feasible, acceptable, and potentially effective approach for improving the mental well-being of caregivers. The intervention demonstrated high feasibility, with a recruitment rate of 91.6% and 100% attendance, completion, and retention. These metrics suggest that the 18-day program was appealing to caregivers and did not place an undue burden on participants.

Acceptability was also high. Most caregivers reported that the application was easy to install and use (111/120, 92.5%), helpful for managing stress (98/120, 81.7%), and something they would recommend to other caregivers (96/120, 80%). Although some participants (68/120, 56.7%) acknowledged ignoring notifications at times, most found the number of messages (87/120, 72.5%) and their delivery timing (99/120, 82.5%) to be helpful or appropriate. The mindfulness activities were positively received, with 85% (102/120) of users reporting that they were helpful for both stress management and learning mindfulness.

Regarding effectiveness, pre-post analyses revealed significant reductions in perceived stress, depressive symptoms, and caregiving burden, along with a significant increase in positive aspects of caregiving. Sleep quality also improved significantly. However, the intervention did not produce statistically significant changes in health-related quality of life or trait mindfulness.

### Comparison With Prior Work

Our findings align with prior JITAI research conducted primarily among noncaregiver populations, including smoking cessation and obesity or dietary adherence interventions, as well as mindfulness-triggering approaches in chronic pain [14,16,32]. However, to our knowledge, this is the first study to apply a mindfulness-based JITAI to family caregivers of people with dementia, a group with distinct constraints and stress dynamics that can undermine adherence to traditional MBIs. Most participants reported that the app was easy to use and helpful for learning and applying mindfulness to reduce stress, consistent with proposed mindfulness-based JITAI mechanisms that target negative affect and automatic responding by increasing present-moment attention in real time [14]. Notably, our results suggest that just-in-time delivery is especially well suited for caregivers of people with dementia, who face substantial time constraints due to heavy caregiving duties and other life commitments. By emphasizing brief windows of receptivity, the intervention may facilitate engagement, as reflected in the high attendance and retention rates. Although mindfulness can help caregivers manage stress, dementia care is a long-term trajectory, and traditional MBIs often struggle to achieve sustained impact because of the required time commitment [9]. By targeting receptive moments for self-practice, the JITAI approach shows promise for supporting longer-term practice across the caregiving trajectory.

Prior mindfulness-based JITAIs, such as those targeting substance use, often trigger intervention prompts at moments of vulnerability (eg, cravings) [14]. For caregivers of people with dementia, however, high-vulnerability states (eg, acute stress) can impede practice because stress taxes attentional control, creating cognitive interference that hinders engagement [11]. As such, our brief mindfulness JITAI focused on prompting during receptive moments. To optimize receptivity, we used three complementary approaches: (1) a machine learning–informed static model derived from pilot data, (2) a random-timing model, and (3) a machine learning–based adaptive model that updated using each participant’s receptivity data over time. Overall, most participants rated message frequency and timing favorably, though some notifications were ignored, indicating room for improvement. Because multiple models were deployed concurrently, we cannot isolate the most optimal approach. Future work should directly compare static, random, and adaptive models to maximize receptivity and, in turn, enhance the benefits of mindfulness for this population.

Regarding self-reported outcomes, we observed small to medium reductions in stress and depressive symptoms, consistent with prior MBIs for caregivers of people with dementia [9,24,33]. The mindful coping model posits that decentering disrupts negative thought cycles and supports more positive stress appraisals [10]. Through this mechanism, mindfulness may reduce depressive symptoms by decreasing rumination, which is a repetitive, problem-unresolved focus on negative thoughts [34]. These findings suggest that a mindfulness-based JITAI can yield benefits that align with traditional MBIs while requiring a less intensive and more flexible structure than standard 8-week programs. Such a less intensive approach may reduce burden and improve accessibility by delivering support via a mobile app, thereby enhancing reach for more caregivers. However, because we did not include a traditional MBI control, we cannot directly compare effect sizes on psychosocial outcomes. Future research should compare JITAI-based mindfulness interventions with standard, group-based mindfulness interventions to clarify their relative efficacy in reducing stress and depressive symptoms among caregivers of people with dementia.

We also observed a small reduction in caregiving burden, diverging from findings in previous trials of MBIs for caregivers of people with dementia that often show no effect on caregiving burden [24,33]. One explanation is that self-directed practices and face-to-face attendance in traditional MBIs add to already busy schedules and are frequently affected by low adherence to self-directed practice. If caregivers perceive the intervention as too intensive, they may lack the motivation to engage in self-practice; yet informal mindfulness practice has been linked to improvements in psychological well-being [35]. By delivering prompts when caregivers are most receptive, a just-in-time approach may better motivate self-directed practices. Heightened moment-to-moment attention in real time via a JITAI may also foster more positive appraisals of caregiving experiences, aligning with our observed gains in positive aspects of caregiving, an outcome rarely examined in prior MBI trials in this population. Cross-sectional evidence similarly links mindfulness with more positive caregiving experiences [36]. Consistent with reduced burden, we also found improvements in subjective sleep quality, which contrasts with some prior findings from MBIs in this population [37,38]. A recent scoping review supports an association between caregiving burden and subjective sleep quality [39]. Because behavioral and psychological symptoms of dementia are often a major source of burden [40], delivering mindfulness via a JITAI with increased self-practice may facilitate caregivers’ translation of mindfulness into more positive responses to behavioral and psychological symptoms of dementia, potentially reducing burden [9]. Given the absence of a control group, these findings should be interpreted cautiously and not as evidence of causality. Randomized controlled trials (RCTs) with process evaluations are needed to elucidate mechanisms underlying reductions in caregiver burden in mindfulness-based JITAIs for this population.

In contrast, neither trait mindfulness nor health-related quality of life improved after the brief mindfulness–based JITAI. This suggests that the 18-day dosage, including 4 days of psychoeducation, may be insufficient to shift dispositional mindfulness, which typically requires sustained practice over a longer period [41]. For example, longer programs (eg, 16-wk formats) have shown gains in trait mindfulness in caregivers of people with dementia [23]. This may also explain the absence of improvement in health-related quality of life, given that trait mindfulness can mediate the relationship between stress and health-related quality of life [42]. Future JITAI-based mindfulness interventions should systematically examine dose-response relationships to clarify their effects on trait mindfulness in this population.

### Implications

This study demonstrates the feasibility and preliminary effectiveness of a mindfulness-based JITAI to support family caregivers of people with dementia. By prompting brief mindfulness micropractices at moments of higher receptivity, the intervention directly addresses practical barriers that commonly limit caregivers’ engagement with traditional MBIs (eg, time constraints, competing care demands, and difficulty sustaining self-directed practice). If these findings are confirmed in controlled trials, this delivery approach could be implemented as a low-intensity, scalable adjunct to the existing community and nongovernmental organization caregiver services by providing between-session support, extending reach to caregivers who are homebound or unable to attend groups regularly, and improving service efficiency through engagement data that can help identify caregivers who may benefit from additional dose, booster support, or stepped-care escalation. In this way, a mindfulness JITAI may strengthen routine caregiver services by enhancing access, personalization, and continuity of support with minimal additional manpower burden to the health and community care system.

### Limitations

This study has several limitations. First, the pre-post, quasi-experimental design lacks a control group, precluding causal inference. Although we observed pre-post reductions in perceived stress and other psychosocial outcomes, these changes cannot be attributed solely to the brief mindfulness JITAI. We encourage future studies to evaluate the program using more rigorous research designs, such as RCTs. Second, we did not include process indicators to examine mechanisms of action; thus, we cannot confirm that improvements resulted from enhanced self-directed mindfulness practices. Future studies should incorporate process measures and mediation analyses to elucidate the mechanisms of change in JITAIs and clarify their unique benefits relative to traditional delivery. Third, although our goal was to examine the general effects of a JITAI, the optimal delivery algorithm remains unclear. Future trials may consider using a microrandomized trial design to test whether a static, random, or adaptive model most effectively predicts and acts upon moments of receptivity. Fourth, this study examines only the effects of the brief JITAI on caregivers. However, a recent meta-analysis highlights dyadic psychosocial effects, whereby depressive symptoms in people with dementia can negatively affect caregivers’ quality of life [43]. Given the close linkage between caregivers’ and care recipients’ psychosocial health, future research should evaluate the effects of the brief mindfulness JITAI at the dyadic level. Fifth, the study’s generalizability may be affected by the sample characteristics and the technology eligibility criteria. In our sample, participants were relatively young (mean age of 45.4, SD 11.8 y), predominantly female (80/120, 66.7%), and highly educated (66/120, 55% tertiary education), whereas territory-level data on carers of older people overall suggest an older caregiver profile (74.5% female among 966 respondents; mean age of 54.5, SD 18.1 y) [44]. Due to the development and resource constraints in implementing the intervention on both operating systems in parallel, the study was delivered on Android only, and caregivers who primarily used iOS devices were excluded. This platform restriction may have introduced selection bias toward more digitally ready participants and may partly explain the relatively younger and more educated sample. In addition, higher educational attainment may reflect self-selection (eg, greater health literacy or confidence with app-based, self-guided interventions), potentially inflating feasibility, acceptability, and engagement estimates. Future trials should broaden recruitment channels and support multiplatform delivery, alongside enhanced onboarding and technical support, to better capture older, male, and lower-literacy caregiver subgroups. Sixth, the intervention was brief, and there was no long-term follow-up; thus, the sustainability of effects remains unclear. The program lasted 18 days, and outcomes were assessed only immediately post intervention, limiting conclusions about whether observed improvements would be maintained after prompts ended. Given that dementia caregiving is a long-term role, future studies should include longer follow-up (eg, 1-3‐6 mo) to evaluate durability and to determine whether maintenance supports (eg, tapered prompting, booster sessions, or continued low-intensity prompting) are required to sustain practice and benefits under real-world caregiving conditions.

### Conclusions

A brief, mindfulness-based JITAI appears feasible and highly acceptable for supporting caregivers of people with dementia. We observed statistically significant reductions in perceived stress, depressive symptoms, and caregiving burden, alongside increased positive aspects of caregiving and subjective sleep quality, supporting the potential of adaptive, technology-enabled interventions to provide timely, personalized support tailored to caregivers’ complex needs. Future work should involve a rigorously designed, adequately powered RCT to confirm efficacy, clarify mechanisms, and inform broader implementation.

## Supplementary material

10.2196/87316 Checklist 1 CONSORT checklist.

## Abbreviations

CONSORT: Consolidated Standards of Reporting Trials
JITAI: just-in-time adaptive intervention
MBI: mindfulness-based intervention
MBSR: mindfulness-based stress reduction
PAC: Positive Aspects of Caregiving
PSS-14: 14-item Perceived Stress Scale
RCT: randomized controlled trial
